# Management of gestational weight gain in obese or overweight women based on resting energy expenditure: A pilot cohort study

**DOI:** 10.1097/MD.0000000000031683

**Published:** 2022-12-09

**Authors:** Xiuling Zhao, Shi Wu Wen, Wei Ma, Pili Xu, Chunmei Zhang, Shan Jiang, Laura M. Gaudet, Jie Gao

**Affiliations:** a Department of Obstetrics and Gynecology, Beijing LuHe hospital, Capital Medical University, Beijing, China; b Clinical Epidemiology Program, Ottawa Health Research institute, Ottawa, Canada; c Department of Obstetrics and Gynecology, University of Ottawa, Ottawa, Canada; d School of Epidemiology and Public Health, University of Ottawa, Ottawa, Canada; e Department of Nutrition, Beijing LuHe hospital, Capital Medical University, Beijing, China; f Department of Obstetrics and Gynecology, Queen’s University, Kingston, Canada.

**Keywords:** diet, obesity, overweight, pregnancy, total energy expenditure, weight gain

## Abstract

Resting energy expenditure (REE) comprises 60% of total energy expenditure and variations may be associated with gestational weight gain (GWG). This study aims to explore the usability and feasibility of REE guided intervention for GWG in obese and overweight women. We conducted a prospective cohort study in LuHe Hospital of Capital Medical University in Beijing, China between May 1, 2017 and May 31, 2018. Obese/overweight women who had routine prenatal care visit at 10 to 13 weeks of gestation, were recruited after written informed consent was obtained. The intervention group (those women who were recruited between January 1 and May 31, 2018) used REE calculated daily total energy to manage GWG, while the control group (those women who were recruited between May 1 and December 31, 2017) used prepregnancy body mass index calculated daily total energy to manage GWG. GWG and daily total energy between the 2 groups were recorded from 10 to 13 weeks of gestation to delivery. A total of 68 eligible women (35 in intervention group and 33 in control group) were included in the final analysis. Daily total energy in the intervention group increased less than the control group, especially from 2nd trimester to 3rd trimester (1929.54 kcal/d vs. 2138.33 kcal/d). The variation of daily total energy from 1st trimester to 3rd trimester in the intervention group was lower than the control group (226.17 kcal/d vs 439.44 kcal/d). Overall GWG of the intervention group (13.45 kg) was significantly lower than the control group (18.20 kg). The percentage of excess-GWG in the intervention group (31.42%) was also significantly lower than the control (57.57%). Findings from our pilot study suggest that diet recommendation basting on REE may improve management of GWG in obese/overweight women.

## 1. Introduction

Obesity/overweight is a major public health problem worldwide. In 2015, rates of obesity and overweight in Chinese adults were 11.9% and 30.1%, respectively, and about 50% women were obese or overweight at the beginning of their pregnancy.^[1,2]^ In 2009, the Institute of Medicine recommended that overweight women gain 7 to 11.5 kg during pregnancy, and obese women limit gestational weight gain (GWG) to 5 to 9 kg.^[3]^ The Institute of Medicine weight gain guidelines have been widely accepted. Yet a systematic review in 2017, the authors found that about 47% of women gained excess weight than the recommended weight.^[4]^ Excessive GWG is associated with a multitude of complications that impact both maternal and neonatal health. These adverse short- and long-term health consequences include preeclampsia, gestational diabetes mellitus (GDM), postpartum weight retention, macrosomia, neonatal hypoglycemia, and admittance to the neonatal intensive care unit.^[4–7]^ A recent review of about 15,581 women in Beijing, China showed that 67.8% of prepregnant obese women had excess weight gain during pregnancy.^[8]^

GWG is closely related to total energy expenditure (TEE), and TEE of pregnant women with obesity/overweight varied greatly. It remains controversial regarding the ideal method to monitor/manage total energy during pregnancy.^[9,10]^ The most widely used method is based on pre-pregnant body mass index (BMI), although this method often leads to an overestimation of the caloric needs in obesity/overweight women.^[9]^ An excess of caloric intake leads to hyperglycemia with increased CO_2_, free fatty acid, and excess GWG.^[11]^ Alves et al suggested that indirect calorimetry should be performed preferentially in pregnant women with obesity/overweight to determine TEE adequately, to adjust for over- or under-energy intake.^[9]^

Previous recommendations for obese women consist of assessment and counseling, which includes the provision of information on the increased health risks of obesity in pregnancy and encouragement to make lifestyle changes to support weight management. Unfortunately, to date, lifestyle intervention trial had limited success at mitigating excess-GWG.^[12,13]^ It is well known that pregnant women usually reduce their physical activities and expend less energy, especially for obese/overweight women.^[14]^ Therefore, physical activity is not the main contributor of TEE in pregnant women.^[15]^ Increased basal metabolic rate (BMR) is instead one of the major components of TEE during pregnancy.^[16]^ Resting energy expenditure (REE) and BMR are interchangeable terms,^[17]^ and REE is frequently used in clinical practice due to difficulties in the measurement of BMR. An important concept in energy balance is that TEE is composed primarily of REE, which accounts for approximately 60% to 70% of TEE.^[18]^ REE shows a significant correlation with energy intake (EI).^[10,19]^ Weight and body fat are important factors contributing to variability of REE during pregnancy and are correlated to cumulative REE in pregnancy. Recently, studies have found that changes of REE reflect increase in calorie needs and weight gain.^[18,20]^ Some investigators hypothesized that interventions could be developed to tailor recommendations for EI for women’s needs based on REE measured in clinical setting, especially for obese/overweight women.^[18,21,22]^ We hypothesized that REE could be improve the management of GWG. We therefore explored the usability and feasibility of using REE guided diet recommendations to manage GWG for pregnant women with obesity/overweight in a small-scale study in China.

## 2. Methods

### 2.1. Study design and ethics

This was a prospective cohort study, and was conducted in accordance with the Strengthening the reporting of cohort Study in STROBE guidelines. This study complies with the Declaration of Helsinki, for ethical considerations. We obtained approval from the Research Ethics Committee of LuHe Hospital of Capital Medical University in Beijing (2018-LHKY-027-02), China, before the commencement of this study, and obtained written informed consent from all participating patients.

### 2.2. Study population

We recruited pregnant women with obese (BMI, weight in kg/height in M^2^ ≥ 30) or overweight (30 > BMI ≥ 25) who visited LuHe Hospital, Capital Medical University in Beijing, China, for their routine prenatal care at 10-13 weeks of gestation between May 1, 2017 and May 31, 2018. High-risk pregnancies with the following diseases were excluded: multiple gestations, fetal growth problems, gastrointestinal disorders, malabsorptive diseases, hyper- or hypo-parathyroid conditions, diabetes mellitus, cardiac diseases and conditions, and intellectual disabilities.

### 2.3. REE and TEE calculations

For the intervention group, REE was measured at recruitment (1st trimester 10–13 weeks of gestation) and then again at 2nd trimester (24–28 weeks of gestation) and 3rd trimester (32–36 Weeks of gestation). For this group, TEE was obtained by measuring REE and physical activity index (PAI). TEE recommendation was adjusted, depending on REE from 2nd trimester to 3rd trimester.

Below is the formula for TEE calculation for the 2 groups:

Intervention group:

TEE = REE × PAI^[23]^

PAI was assessed using the International Physical Activity Questionnaire (IPAQ) before measuring REE. IPAQ reported as Metabolic Equivalent-Minutes per week (MET-minutes/week). PAI was obtained at recruitment (1st trimester 10–13 weeks of gestation) and then again at 2nd trimester (24–28 weeks of gestation) and 3rd trimester (32–36 weeks of gestation).

Exercise level was defined as the following:

Low level (PAI = 1.2): total physical activity of MET-minutes/week < 600.

Moderate level (PAI = 1.5): total physical activity of MET-minutes/week between 600 and 3000.

High level (PAI = 1.7): total physical activity of MET-minutes/week > 3000^.[3, 23]^

### 2.4. REE

We used Vmax Encore 29n (product of CareFusion company USA) to measure REE. The measurement was conducted by a professional technician, at LuHe Hospital Adult Nutrition Research Center. Participants were asked to arrive at the nutrition center in the morning after a 12-hour overnight fast. They were instructed to have a 30-minute rest in a semi-recumbent position, remaining calm during this time. They were then escorted to a comfortable (temperature maintained at 21–22°C) room and asked to lie down quietly for 30 minutes. An instrument with a transparent plastic hood was positioned to cover the area from head to neck, with fresh air free flowing through the instrument. After the equilibration period, oxygen consumption (V_O2_) and carbon dioxide production (V_CO2_) were monitored continuously for 30 minutes while the participant remained awake, silent, and motionless. Minute by minute values of V_O2_, V_CO2,_ respiratory quotient, and energy expenditure were monitored over the 30-minute collection period to obtain the REE. REE and body composition were measured at recruitment (1st trimester, 10–13 weeks of gestation), 2nd trimester (24–28 weeks of gestation), and 3rd trimester (32–36 weeks of gestation).

REE = (3.941 × V_O2_ + 1.106 × V_CO2_) × 4.18(Weir’s)

**For the control group:**

TEE = (Height-105)_Kg_ × EC^[24]^

EC: energy coefficient according to the level of Pre-pregnant BMI.

Overweight: 25–30 Cal/kg·day

Obese: 20–25 Cal/kg·day

Ideal Weight (Kg) = Height-105

**Nutritional recommendation**

The hospital nutritionist made dietary recommendations based on TEE by the way of Food Exchange Share. For the intervention group, daily total energy recommendations were adjusted, depending on REE measured at 2nd trimester and 3rd trimester. For the control group, daily total energy was calculated at recruitment by the standard method involving ideal weight and pre-pregnant BMI only. An extra 300 Kcal was added to the daily total energy at 2nd trimester, and an extra 450 Kcal was added at 3rd trimester as recommended by guideline for the control group.^[3,25]^ GWG of 2 groups were monitored and recorded each week. Dietary recommendations for the 2 groups were based on guidelines of GDM. GDM guidelines were used because no dietary guideline existed specifically for pregnant women who were obese/overweight.^[26]^ Daily total energy was generally provided by approximately 50% to 60% of energy from carbohydrate, 25% to 30% from fat, and 15% to 20% from protein, divided into 3 meals and 3 snacks.^[27]^ Food diary was recorded on daily basis by the participants themselves, including carbohydrate, meat, vegetables, and oil. Food was weighted with an electronic scale with a precision to 1 g. Study staff frequently contacted participants by telephone, text messages or WeChat to check compliance every week. Food diary provided a basis for estimating total EI to nutritionist. It was considered satisfactory if total EI was not less or more than 10% to 15% of the calculated TEE; otherwise, the nutritionist would give them dietary instruction.

#### 2.4.1. Anthropometrics data.

Anthropometrics data were collected at every study visit for both groups except for prepregnancy weight which was obtained by participants recall at first study visit. Research staff collected height using an anthropometer with a precision of 1 mm for height. These measurements were used to calculate BMI as kg/m^2^. During the duration of the study, maternal weight was measured with an electronic scale with precision to 0.1 kg at each study visit. Participant was asked to wear a gown of known weight and with no shoes or jewelry on. Rate of weight gain was calculated as the total weight gain in kilograms divided by the number of weeks the participant was followed.

#### 2.4.2. Laboratory procedures.

Venous blood was drawn from study participants after a 12-hour fast, as well as plasma and serum samples. The plasma and serum samples were stored at ≥80°C before testing. Analyses of triglycerides (TG), total cholesterol (TC), high-density lipoproteins cholesterol (HDL-C), and low-density lipoproteins cholesterol (LDL-C) were conducted at the Biochemistry Laboratory of LuHe Hospital. TG was detected by phosphor glycerol oxidase method. TC was measured with bile Sterol oxidase method. HDL-C and LDL-C was detected by direct one-step method. Reagent from North Provided by Beijing Jiuqiang company, Olympus AU400 automatic biochemical analyzer (OLYMPUS OPTICAL Co.Ltd, Japan) was used for testing. When TG more than 1.7 mmol/L or/and TC higher than 5.17 mmol/L or/and HDL-C lower than 0.77 mmol/L or/and LDL-C higher than 4.13 mmol/L were abnormal.

#### 2.4.3. Demographic and clinical data collection.

Demographic and clinical data including occupation, age, education, family income, family history, past history, gravidity, gestational age, and pregnancy complications were collected by study staff using an ad hoc questionnaire developed by the research team. Gestational age was based on last date of normal menstrual period and validated by ultrasound examination in early gestation. Study staff assessed exercise condition by the IPAQ to estimate PAI.

### 2.5. Statistical analysis

Statistical analysis was performed using the SPSS 18.0 (SPSS, Inc, Chicago, IL) for Windows. Distribution of data was assessed by Kolmogorov–Smirnov’s test. If the *P*-value of the Kolmogorov–Smirnov’s test was higher than .05, the data was considered normally distributed and 2 independent samples Student’s *t*-test (2-tailed) was used to compare the mean differences between the 2 study groups. For categorical data, the differences between the 2 groups were compared by using the Pearson χ^2^ or Fisher’s exact test. All test statistical tests were 2-sided, with significance evaluated at *P* < .05.

## 3. Results

**Figure 1.**
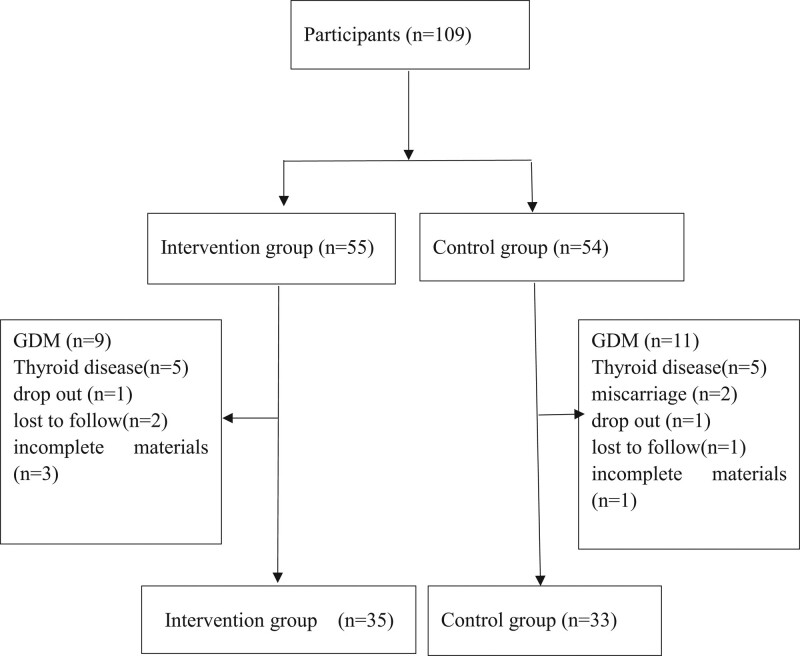
Flow diagram of inclusion and attrition rates in the current study. GDM = gestational diabetes mellitus.

A total of 109 obesity/overweight women were recruited into this study. They were divided into 2 groups according to the study periods. The intervention group (n = 55) were recruited between January 1 and May 31, 2018. The control group (n = 54) were recruited between May 1 and December 31, 2017. Forty-one participates were excluded the study for the following reasons: diagnosed GDM (9 in the intervention group, 11 in the control group), thyroid disease (5 in the intervention group, 5 in the control group), miscarriage (2 in the control group), drop out trail (1 in the intervention group, 1 in the control group), lost to follow (2 in the intervention group, 1 in the control group), or with incomplete study data (3 in the intervention group, 1 in the control group). A total of 68 eligible women (35 in the intervention group n = 35 and the control group n = 33) were included in the final analysis (Fig. 1).

### 3.1. Comparison of baseline characteristics between the 2 groups

Table 1 compares baseline demographic and clinical characteristics between the 2 study groups. There was not statistical difference in age, BMI, gravidity, parity, education, and family income between the 2 groups. All of these women delivered infants weighing normally between the 10th and 90th percentile according to gestational age. There was no difference between 2 groups in the concentrations of TG, TC, HDL-C, and LDL-C at recruitment either.

**Table 1:** Baseline demographic and clinical characteristics data.

Item	Intervention group (N = 35)	Control group (N = 33)	*P* value
Maternal age (SD) (yr)	32.83 (5.39)	31.83 (3.85)	.49
Body mass index prepregnant (SD) (kg/m^2^)	29.97 (4.58)	28.30 (3.53)	.18
Gravidity (N)			
1	13	11	.76
2	10	12	
≥3	12	10	
Parity (N)			
1	20	25	.86
2	15	8	
Maternal education			
<12 yr of schooling	0	0	
≥ 12 yr of schooling	13	15	.48
University/college or above	22	18	
Family income (¥)/mo			
Less than 10000	8	9	.89
10000–19999	15	14	
20000 or greater	12	10	
Gestational age at delivery (SD) (wk)	37.59 (4.60)	38.36 (3.60)	.54
Mean birth weight (SD) (kg)	3.27 (0.53)	3.43 (0.60)	.33
plasma lipid concentration (SD) (mmol/L)			
Triglycerides	1.79 (0.81)	1.60 (0.87)	.23
Total cholesterol	4.56 (0.73)	4.99 (1.06)	.15
HDL-C	1.61 (0.28)	1.78 (0.52)	.22
LDL-C	2.53 (0.58)	2.67 (0.77)	.49

Data are listed as mean (SD); Qualitative variables are expressed as n (%). Independent *t* test were performed for all continuous variables to determine whether there were statistically significant differences between 2 groups, and means (SD) are reported. Chi-square tests were evaluated for all baseline categorical variables to determine whether there were statistically significant differences between groups.

HDL-C = high-density lipoproteins cholesterol; kg/m^2^ = weight/height^2^; LDL-C = low-density lipoproteins cholesterol; N = number; SD = standard deviation.

### 3.2. Data of REE and TEE

Table 2 presents data on REE and TEE. REE increased from 1228.20 ± 183.12 kcal/d in the first trimester up to 1607.09 ± 406.35 kcal/d in the third trimester. TEE of intervention group showed a similar rising trend, from 1754.28 ± 166.18 kcal/d to 1929.54 ± 366.18 kcal/d. For the control group, TEE increased from 1688.33 ± 131.69 kcal/d to 2160.33 ± 151.69 kcal/d. The change of TEE in the intervention group (226.17 kcal/d) was significantly lower than in the control group (439.44 kcal/d).

**Table 2:** Data of resting energy expenditure (REE) and total energy expenditure (TEE).

item	1st trimester	2nd trimester	3rd trimester	ΔTEE (kcal/d)
Intervention group				-
REE (kcal/d)	1228.20 (183.12)	1521.15 (256.93)	1607.09 (406.35)	-
PAI	1.42 (0.91)	1.20 (0.09)	1.21 (0.32)	-
TEE (kcal/d)	1754.28 (166.18)	1826.17 (282.62)	1929.54 (366.18)	226.17 (70.92)
Control group				
TEE (kcal/d)	1688.33 (131.69)	1958.33 (101.60)	2160.33 (151.69)	439.44 (132.80)
*P* value	.21	.47	.02	.03

Independent samples *t* test were performed for all normally distributed data and mean (SD) are reported. ΔTEE means the change of TEE in pregnancy.

kcal = kilocalories; PAI = physical activity index; REE = resting energy expenditure; SD = standard deviation; TEE = total energy expenditure.

### 3.3. Intervention effect on GWg

Table 3 summarizes the results of GWG. Both groups experienced similar mean GWG during 1st trimester and 2nd trimester. In 3rd trimester, mean GWG in the intervention group (0.47 kg/wk) was significantly lower than that of in the control group (0.89 kg/wk). The rate of excess-GWG in the intervention group (31.42%) was also significantly lower that of in the control group (57.57%).

**Table 3:** Intervention effect on gestational weight gain (GWG) data.

item	Intervention group N = 35	Control group N = 33	P-value
Mean GWG (SD) (kg/wk)			
1st trimester	0.26 (0.16)	0.27 (0.17)	0.90
2nd trimester	0.77 (0.30)	0.65 (0.38)	0.09
3rd trimester	0.47 (0.53)	0.89 (0.69)	0.00
Overall GWG (SD) (kg)	13.45 (4.16)	18.20 (4.85)	0.03
Categories of GWG (%)			
Under	2 (5.71)	1 (3.03)	0.58
Up to	22 (62.85)	13 (39.39)	0.05
Excess	11 (31.42)	19 (57.57)	0.03

Categories of GWG was based on the Institute of Medicine (IOM) recommended GWG guideline. Data are listed as mean (SD); Qualitative variables are expressed as n (%). Independent *t* test were performed for all continuous variables to determine whether there were statistically significant differences between 2 groups, and means (SD) are reported. Chi-square tests were evaluated for all baseline categorical variables to determine whether there were statistically significant differences between groups.

GWG = gestational weight gain; N = number; SD = standard deviation.

## 4. Discussion

The results from our pilot study suggest that it may be practical to use REE-based diet recommendation to manage GWG for obesity/overweight women during pregnancy. Our study also found that in the 3rd trimester, mean GWG in the intervention group was significantly lower than the control group. However, no difference in GWG was observed during 1st trimester and 2nd trimester. It may be a long process to change the dietary habits with improved GWG for obese/overweight women.

Excess EI during pregnancy may have a negative impact on health outcomes for pregnant women and their infants.^[26]^ GWG is closely related to TEE and TEE of pregnant women with obesity/overweight varied.^[18]^ A recent meta-analysis of 32 studies showed that TEE increased by 84 to 363 kcal between early and mid and by 179 to 682 kcal between early and late pregnancy.^[18]^ Half of the studies that measured TEE reported increases that were below the EI recommendations of an additional 340 and 450 kcal/d in the 2nd and 3rd trimesters, respectively.^[10,22,28]^ Our study data showed that TEE increased 226.17 kcal/d in the intervention group, significantly lower than the control group (439.44 kcal/d) in the whole pregnancy. Obviously, it is inappropriate to adopt a “one size fits all” energy recommendation for women, leads to excess- or under- weight gain in pregnancy. Our study suggested that in order to get appropriate weight gain for obesity/overweight women, individual EI recommendation is needed for women who have more risks of excessive GWG.

Physical activity is not the main contributor of TEE in pregnant women, while REE is one of the major components (about 60%–70%) of energy expenditures during pregnancy.^[16]^ REE rises ranging from 13% to 35% in pregnancy, because of increased maternal body mass.^[18,29]^ Rasmussen hypothesized that nutritional intervention should be developed to tailor recommendations for EI for obese/overweight women’s needs based on REE measured in clinical setting.^[21]^ Our pilot study, basing on REE to guide diet to manage GWG, aims to test this hypothesis. Considering increased REE or BMR, we chose the formula combine REE and PAI, to calculate TEE. Our study suggested that individual EI recommendation is needed for obesity/overweight women to get appropriate weight gain. Whether this formula fits other categories pre-pregnancy BMI, requires conformation by additional studies.

### 4.1. Strengths and limitations

To the best of our knowledge, this is the first study in China and one of the few studies in the world that has assessed the effect of REE calculated TEE on managing GWG. The study generated results that could be useful in the management of GWG for obese/overweight women. However, this study has several limitations. First, because of resources and other logistic difficulties in our hospital, a before-after design instead of a parallel randomized controlled trial conducted, making it difficult to make a direct estimation of the effect of REE based management of GWG with routine care. On the other hand, before-after comparison avoided communication among patients in the intervention group and the control group with reduced risk of cross-contamination. There is no change in maternity care team and hospital facilities, and the distribution of baseline characteristics of the 2 groups was essentially the same. As a result, the risk of bias/confounding from non-randomized design may be small. Second, this study was based on a sample size recruited in a single center. Replication of the study findings by future large-scale studies is needed. Third, some of the data such as pre-pregnancy weight was collected based on women’s recall, which may introduce certain amount of bias.

## 5. Conclusion

Our pilot study suggests that diet recommendation based on REE may improve management of GWG for obese/overweight women. Large-scale randomized controlled trials are needed to confirm the findings from our pilot study.

## Authors contributions

X.Z. and J.G. designed the study, conducted the statistical analysis of the data, and drafted the manuscript. W.M and P.X. conducted the lab tests and collected the clinical data. C.Z and S.J performed nutritional and lab analysis. L.G. reviewed the interpretation of the result and the manuscript. S.W.W supervised the study implementation and statistical analysis, and critically revised the manuscript. All authors participated in the review and critical revisions of the final manuscript. The correspondence authors attest that all listed authors meet authorship criteria and that no others meeting the criteria have been omitted.

**Data curation:** Xiuling Zhao, Shan Jiang.

**Formal analysis:** Xiuling Zhao, Shi Wu Wen, Wei Ma, Jie Gao.

**Investigation:** Xiuling Zhao, Pili Xu, Chunmei Zhang.

**Methodology:** Shi Wu Wen, Laura M. Gaudet.

**Writing—original draft:** Xiuling Zhao.

**Writing—review and editing:** Wei Ma.
